# The Mycobiota of High Altitude Pear Orchards Soil in Colombia

**DOI:** 10.3390/biology10101002

**Published:** 2021-10-05

**Authors:** Lidia Nicola, Angela Yaneth Landínez-Torres, Francesco Zambuto, Enrica Capelli, Solveig Tosi

**Affiliations:** 1 Laboratory of Mycology, Department of Earth & Environmental Sciences, University of Pavia, 27100 Pavia, Italy; francescozambuto@hotmail.it (F.Z.); enrica.capelli@unipv.it (E.C.); solveig.tosi@unipv.it (S.T.); 2 Faculty of Agricultural & Environmental Sciences, Juan de Castellanos University, 150001 Tunja, Colombia

**Keywords:** soil, fungi, biodiversity, metabarcoding, Colombia, South America, *Mortierella*

## Abstract

**Simple Summary:**

Soil fungi are extremely important in the agro-environment. They are among the main decomposers of organic matter, contributing to carbon, nitrogen, and phosphorous cycles. They often establish positive relationships with plants, protecting them from pathogens and abiotic stresses. This study aimed to uncover the soil fungal communities of two high altitude pear orchards with biomolecular techniques. We found a rich and diverse assemblage, dominated by fungi belonging to Ascomycota and Mortierellomycota. Most of the found species were novel records for soil fungi in Colombia. The most common fungal genera were *Mortierella*, *Fusarium*, *Pseudaleria* and *Cylindrocarpon*. Among the identified fungi, some species are known to be bioactive, with promising activities as biocontrol agents, plant-growth promoters, and producers of valuable substances. These results could contribute for a more attentive management of Colombian pear orchards in future and an enrichment of knowledge on Colombian biodiversity.

**Abstract:**

In Colombia, the cultivation of deciduous fruit trees such as pear is expanding for socio-economic reasons and is becoming more and more important for the local population. Since organized cultivation is slowly replacing sustenance cultivation, scientific information on the present agro-environment is needed to proceed in this change in an organic and environmentally friendly way. In particular, this study is an accurate description of the mycobiota present in the bulk soil of two different high altitude pear orchards in the Colombian Andes. The metabarcoding of soil samples allowed an in-depth analysis of the whole fungal community. The fungal assemblage was generally dominated by Ascomycota and secondly by Mortierellomycota. As observed in other studies in Colombia, the genus *Mortierella* was found to be especially abundant. The soil of the different pear orchards appeared to host quite different fungal communities according to the soil physico-chemical properties. The common mycobiota contained 35 fungal species, including several species of *Mortierella*, *Humicola*, *Solicoccozyma* and *Exophiala*. Moreover, most of the identified fungal species (79%) were recorded for the first time in Colombian soils, thus adding important information on soil biodiversity regarding both Colombia and pear orchards.

## 1. Introduction

Agriculture is a sector of upmost importance all over the world. Globally, agricultural land use is 38% of the global land surface and Colombia devotes approximately 44 million hectares to agricultural use [1]. The economy of the Colombian department Boyacá is mainly based on agricultural and livestock production. In this region, mainly sustenance crops are grown (potato and onion), but fruit crops occupy a key place for economic and social reasons. In fact, the living standards of fruit growers is above that of producers of other food crops. Due to its extremely differentiated landscape in altitude, up to 36 different species of fruit trees are cultivated in Boyacá. Thus, the department of Boyacá is first in the national production of fruit crops, with Nuevo Colón, also called the “Fruit Garden of Colombia”, a particularly representative municipality for national fruit production. Four species (apple, pear, plum, and peach) are cultivated in a large range of varieties that have successfully adapted to the agro-ecological conditions of the region and, as they are preferred internally for their quality and freshness, compete favorably with imported fruits on the market [2,3,4]. Usually, deciduous fruit trees of different species and varieties are cultivated in small farms (less than three hectares), in communities with little or no irrigation infrastructure, and with little specialized technical assistance. However, over the years, some advances have been made in terms of business management, introduction of new varieties, technological innovations, irrigation infrastructure, associative work mentality, larger farms and crop planning [3,4]. Project Deciduous of the program of Agroecology of the University “Fundación Universitaria Juan de Castellanos” (JDC, Tunja, Colombia) is working in this direction in the municipality of Soracá. This project focuses on the cultivation of deciduous fruit trees as a part of the development of new production for the area [5], and it is based at the experimental farm San Isidro Labrador of the JDC, located in Soracá. The JDC Deciduous Project currently has about 3300 trees planted on an area of 10 hectares at an altitude of 2840 m.a.s.l. and studies the cultivation of deciduous fruit trees such as apple, pear, peach, and prune trees. This project has scientific aims, such as the genetic improvement and molecular characterization of crops, as well social goals, such as educating the farmers of the area, familiar only with sustenance agriculture, on good agricultural practices for deciduous fruit trees.

A careful and environmentally respectful management of agro-resources, such as soil, is fundamental for the sustainable development of a country, especially with the sociological and economic situation found in the Boyacá department (Colombia). Studying not only the physico-chemical and pedological characteristics of a cultivable soil, but also the microorganisms in the area can help find the most appropriate ways to manage that soil. The entire fungal assemblage, also known as mycobiota, plays a key role in soil, particularly in natural carbon, nitrogen, and phosphorous cycles [6]. In fact, fungi are fundamental for the decomposition of organic matter. Moreover, some species, thanks to their specific activity, can bio-fertilize soil, enhance plant growth and productivity and antagonize plant pathogens, working as biocontrol agents. Other species can be, on the other hand, dangerous plant parasites [7]. It is important to remember that the type of soil management, the products used, and tillage can actively shape and change the soil mycobiota [8].

Studies of fungi in Colombia began with Fuhrnman & Mayor [9], whose work focused on parasitic fungi. Later, several studies on fungi were carried out, but attention was aimed mainly at macroscopic fungi and mycorrhizae [10,11,12,13,14,15], leaving the rest of the soil microfungi relatively uncharted. Until 2020, international literature reported approximately 300 different species of soil microfungi in Colombia, with the most abundant genera being *Acaulospora*, *Glomus*, *Penicillium*, *Aspergillus*, *Fusarium* and *Mortierella* [16].

In 2019, almost 24 million tons of pear were harvested worldwide, about 22,000 tons of which were in Colombia [1]. Globally, pear ranks 15th of the most cultivated fruit [17]. Only a few studies focused on the microbiota associated with this crop. Zhang et al. [18] studied how fungal and bacterial communities in pear orchards could be influenced by the presence of intercropping aromatic plants. Other two studies in pear orchards were carried out by Vadkertiová et al. [19], who focused on soil yeasts, and by Huang et al. [20], who studied arbuscular mycorrhizae. Moreover, Zhang et al. [21] studied the rhizosphere bacterial community in pear plants in relation to soil chemical properties. Finally, the fungal communities of other parts of the pear plant were studied, like bark [22] or carpoplane [23].

The aim of the present research was to provide an accurate description of the mycobiota present in the bulk soil of two different high altitude pear orchards in the Colombian Andes, one in a university experimental farm in Soracà and one in a private producer farm in Nuevo Colón. These results will provide a global picture of the soil fungal community associated with this cultivation in Colombia and will be useful for future reference and soil management for experimental farms. Moreover, since little is known on soil fungi in Colombia, our data will contribute to the ongoing process of uncovering Colombian agricultural soil mycobiota.

## 2. Materials and Methods

### 2.1. Area of Study

**Figure 1:**
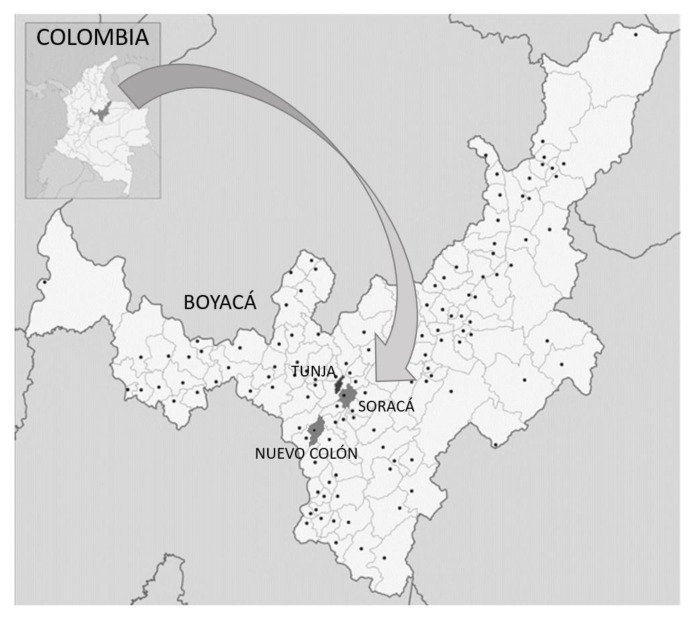
Study area. The municipalities of Soracá and Nuevo Colón in the Department of Boyacá, Colombia, South America. Adapted from Wikimedia Commons [26].

The study area consisted of two different pear orchards at high altitudes in the department of Boyacá, in the Andean region of Colombia (Figure 1). In this region the climate is bimodal, with a rainy season from April to July and a dry season from August to March.

The first pear orchard was located on flat terrain in the experimental farm San Isidro Labrador, belonging to the Fundación Universitaria Juan de Castellanos, in Soracá, department of Boyacá (Colombia, coordinates: 5°30′ N, 73° W, altitude: 2840 m.a.s.l.). The farm is located in a hilly landscape, with a predominance of parental material corresponding to clay-type sedimentary rock alterites [24]. The soil has an umbric epipedon (0-22 cm deep), moist dark brown color (7.5 YR3/2) with slight red mottling (2.5 YR4/8), and clay loam texture (FAr). According to the Holdridge life zones system, the Soracá area is classified as dry mountain forest [24]. The mean annual temperature in this area is 12 °C, with a relative humidity of 70% and average annual precipitation of approximately 750 mm (highest rainfall in May with 111 mm, lowest rainfall in January with 16 mm) [25]. The pear orchard in Soracá (SR) measured at 9111.5 m^2^, the pear variety was Triumph de Vienna and the trees were planted in the spring of 2012. Fertilization was performed with the mineral product Café Producción^®^ (17% N, 6% P, 18% K, 2% trace elements, 0.7 kg per tree) once a year after pruning, usually during February. Weeding was performed by hand with a scythe every two months in the rainy season and every four months in the dry season. To control fungal diseases, a combination of four products was used: Mancozeb 80% (5g/L), Daconil 50C (2.5ml/L), Ossiclor 35WG (5g/L), and Benomil 50 WP Agricense (300g/L).

The second pear orchard was located in a conventional pear cultivation farm of a private producer in Nuevo Colón, department of Boyacá (Colombia, coordinates: 5°21′ N, 73°27′ W, average altitude: 2470 m.a.s.l.). The farm is located in an erosional structural mountain landscape [24]. The soil has an umbric epipedon (0–22 cm deep), wet black color (10YR 2/1), and clay loam texture (FAr). According to the Holdridge life zones system, the Nuevo Colón area is classified as dry mountain forest [24]. The mean annual temperature in this area is 16 °C, with a relative humidity of 80% and average annual precipitation of approximately 900 mm (highest rainfall in July with 124 mm, lowest rainfall in January with 16 mm) [25]. The pear orchard in Nuevo Colón measured 50,000 m^2^, the pear variety was Triumph de Vienna and the trees’ age varied from 28 to 50 years. Fertilization was performed with an organic mixture called “bocachi” (17 tons chicken manure, 6 tons dolime, 6 tons quicklime, 6 tons phosphate rock, 500 kg CaSO_4_·2H_2_O, 4 tons rice husk, 150 kg molasses) and with the chemical fertilizer YaraMila Complex^®^ (5% NO_3_^−^, 7% NH_4_^+^, 11% P_2_O_5_, 18% K_2_O, 2.65% MgO, 19.9% SO_3_, and trace elements) and Nutrimon^®^ (17% N, 6% P, 18% K, 2% trace elements), in an annual dose of 1 kg per tree, usually during the month of March. Weeding was performed with Roundup^®^ Power 2.0 (glyphosate 360 g/L) and Gramoxone^®^ (Paraquat 200 g/L). To control fungal disease Carbendazim, Iprodione and Bellis^®^ were used. The orchard in Nuevo Colón was divided into two parts, one was constituted by a steep slope (60°) at a higher altitude (2568 m.a.s.l., NC-A), while the other one was at the foothill of the former and was constituted by flat terrain at a lower altitude (2540 m.a.s.l, NC-B). In both SR and NC orchards, pear trees were the only cultivated plants.

### 2.2. Sample Collection

Three soil samples for each orchard terrain (three for Soracá SR, three for the NC-A slope in Nuevo Colón, three for the flat part NC-B in Nuevo Colón) were collected during dry season (August 2019), for a total of 9 soil samples. Each soil sample was obtained by mixing three subsamples (20 g each) randomly and aseptically collected along the row at 25 cm from the pear tree and at a depth of 10 cm and put into sterile polyethylene bags. Soil samples were returned to the laboratory in coolers, they were sieved with a 2 mm mesh size, removing roots and plant debris, and they were kept at −20 °C (for metagenomic analyses) or 4 °C (for other analyses) and subsequently used for downstream physico-chemical, fungal charge, and metabarcoding analyses.

### 2.3. Soil Physico-Chemical Analyses and Evaluation of Total Fungal Counts

Physico-chemical properties of soils were determined by the Department of Earth and Environmental Sciences at the University of Milano-Bicocca (Milan, Italy), according to Italian standard protocols (DM 13/09/99). The following parameters were evaluated: pH, organic matter, total nitrogen (N_TOT_), organic carbon (C_ORG_), C/N ratio, plant-available phosphorous (P), calcium (Ca), magnesium (Mg), potassium (K), soil composition in sand, silt, and clay.

Regarding the evaluation of total fungal counts, soil samples were processed within 15 days of collection, using the dilution plate technique [27] to count cultivable microfungi, following the protocol of Landínez-Torres et al. [28]. Four replicates of each sample were prepared, and 100 µL of soil dilution were spread on potato dextrose agar (PDA) plates and incubated at 25 °C in the dark. Inoculated plates were observed continuously over 2 weeks by means of a stereomicroscope, and the number of developed colonies was expressed as colonies forming units (CFU) per gram of soil dry weight.

### 2.4. DNA Extraction, ITS1 Amplification, Illumina Sequencing and Bioinformatic Data Analysis

Total DNA was extracted from 0.5 g of each composite soil sample using the FastDNA^TM^ SPIN Kit for Soil (MP Biomedicals, Santa Ana, CA, USA) according to the manufacturer’s instructions. The extracted DNA was dissolved in 100 mL of DES (DNase/Pyrogen-Free Water), quantified by NanoDrop™ Lite spectrophotometer (Thermo Fisher Scientific Inc.), and stored at −20 °C until PCR amplification. For amplicon production, the ribosomal internal transcribed spacer region 1 (ITS1) was targeted, by using primers BITS and B58S357 [29] linked to Illumina adapters. PCR was performed following the protocol by Landínez-Torres et al. [28]. PCR was performed in a 50-µL volume containing 5 to 10 ng template DNA, 1× HiFi HotStart Ready Mix (Kapa Biosystems, Wilmington, MA, USA), 0.5 µM of each primer. The cycling program, performed on a MJ Mini thermal cycler (Promega corp., Madison, WI, USA), included an initial denaturation (95 °C for 3 min), followed by 25 cycles at 94 °C for 30 s, 58 °C for 30 s, 72 °C for 30 s, and final extension (72 °C for 5 min). PCR amplicons were purified with Agencourt AMPure XP Beads 0.8X (Beckman Coulter, Inc., CA, USA) and amplified following the Nextera XT Index protocol (Illumina, Inc., CA, USA). The purified amplicons were normalized by SequalPrep™ Normalization Plate Kit (Thermo Fisher Scientific Inc.) and multiplexed. The pool was purified with 1X Magnetic Beads Agencourt XP (Beckman Coulter, Inc.) loaded on the MiSeq System (Illumina, Inc.) and sequenced following the V3-300PE strategy. Bioinformatic analysis was performed by Qiime2 version 2020.2 [30]. Raw reads were first trimmed by applying Cutadapt to remove residual primer sequences [31], and then processed with the DADA2 plug-in to perform the denoising step [32]. DADA2 was run with default parameters except for the truncation length: forward and reverse reads were both truncated at the length of 155 nucleotides. The resulting amplicon sequence variant (ASV) sequences were filtered out by applying a 0.05% frequency threshold to discard singletons and very rare sequences. UNITE v.8.2 was used to associate the taxonomy to the remaining ASVs [33], following the classification by Tedersoo et al. [34]. Sequencing and bioinformatic data analysis were performed at BMR Genomics srl (Padua, Italy).

### 2.5. Statistical Analysis

The aim of the statistical analysis performed on our samples was to detect any differences among the pear orchard in the experimental farm (SR) and the two plots of the private fruit producer (NC-A, NC-B). Chemical data and CFUs counts were statistically analyzed with the PAST software package, version 4.03 [35], using the Kruskal–Wallis test with the Bonferroni correction for multiple comparisons. Statistical analysis of the sequencing data was performed with the phyloseq R package, ver. 1.32.0 [36]. To control biasing effects of sequencing depth, samples were rarefied by subsampling to 90% of the depth of the least abundant sample (68,311 sequences). Alpha diversity was calculated using Observed Species, Simpson, and Shannon indices. Pairwise Wilcoxon Rank Sum with Bonferroni correction for multiple testing test was applied to alpha diversity indices to assess any statistically significant differences among orchards. Beta diversity was evaluated using multivariate analysis of the fungal assemblage structure. Specifically, Principal Coordinate Analysis (PCoA) on Bray–Curtis distance matrix was used. To assess any statistically significant difference among the fungal communities in the different orchards, PERMANOVA was used, implemented in the vegan R package, ver. 2.5.6 [37] as the adonis function. To test the OTU differential abundance in the orchards, the DESeq2 R package ver. 1.28.1 was used [38], applying the differential expression analysis based on a negative binomial distribution on non-rarefied data, using a false discovery rate (FDR) cutoff of 0.01.

## 3. Results

### 3.1. Soil Physico-Chemical Analyses and Evaluation of Total Fungal Counts

The soil texture was loam in all the sites (SR, NC-A and NC-B) and the physico-chemical characteristics were quite homogeneous, but two significant differences were detected among the sites (Table 1). Specifically, the soil in the flat part at a lower altitude in the orchard of Nuevo Colón (NC-B) had a significantly higher pH (6.6 ± 0.1), and plant-available phosphorous (281.2 ± 23.6 mg/kg) compared with the soil in the SR orchard (Kruskal–Wallis test, *p* < 0.05).

Regarding the evaluation of total fungal counts, collected SR soil samples showed a significantly higher amount of CFUs (1.4 × 10^6^ CFUs per gram of soil), compared with both NC-A and NC-B samples (6.3 ×10^5^ CFUs and 7.3 × 10^5^ CFUs per gram of soil, respectively; Kruskal–Wallis test, *p* < 0.05). Moreover, in the same Kruskal–Wallis test, the difference in CFUs counts between NC-B and NC-A samples was also statistically significant.

### 3.2. Soil Fungal Assemblage Composition

The sequencing of soil samples on the Illumina MiSeq platform produced a total of 1,822,836 raw reads (approximately 202,537 ± 44,988 per sample). After the filtering, denoising, and merging steps and the elimination of chimeric sequences and rarefaction, 1,223,696 sequences remained (approximately 135,966 ± 39,153 per sample). A total of 629 fungal OTUs were detected (Table S1).

**Figure 2:**
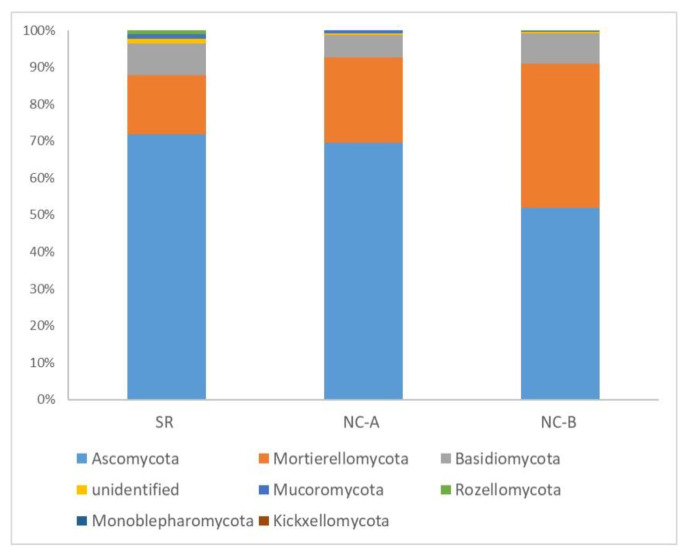
Average soil fungal relative abundances at phylum level of soil samples taken from Soracá (SR) and Nuevo Colón (steep slope plot at higher altitude: NC-A; flat plot at lower altitude: NC-B).

The taxonomic analysis assigned sequences to seven fungal phyla (Figure 2). The fungal assemblage was generally dominated by Ascomycota (64% of total reads), ranging from 72% ± 5% in SR samples to 52% ± 9% in NC-B samples. Mortierellomycota was the second most abundant phylum (27% of total reads), especially numerous in NC-B samples (39% ± 12%). Basidiomycota represented 8% of total reads, ranging from 9% ± 0.2% in SR samples to 6% ± 2% in NC-A samples. OTUs belonging to the phyla Mucoromycota, Rozellomycota, Monoblepharomycota and Kickxellomycota were detected in very low abundances, which was as expected and consistent with the literature (<1% of total reads) [39,40]. The phylum Glomeromycota was not detected in our samples, which was unexpected because pear trees are mycorrhizal [41].

**Figure 3:**
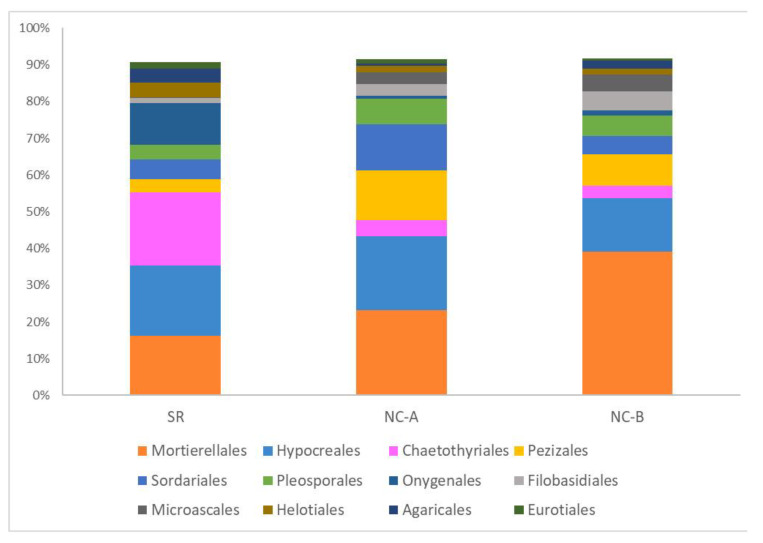
Average soil fungal relative abundances at order level of soil samples taken from Soracá (SR) and Nuevo Colón (steep slope plot at higher altitude: NC-A; flat plot at lower altitude: NC-B). Only the orders with abundances >1% of total reads are plotted in the graph.

At order level, the most abundant one was Mortierellales (Mortierellomycota), ranging from 39% in NC-B samples to 16% in SR samples, followed by Hypocreales (18% of total reads), Chaetothyriales (9% of total reads), Pezizales (8% of total reads) and Sordariales (8% of total reads), all belonging to the Ascomycota phylum (Figure 3).

The genus *Mortierella* was by far the most represented one, with a general abundance of 27%, followed by *Fusarium* (6%), *Pseudaleuria* (5%), *Cylindrocarpon* (4%), *Solicoccozyma* (3%), *Humicola* (3%), and *Exophiala* (3%).

The alpha diversity found in the fungal communities of the three plots (SR, NC-A and NC-B) was similar and there were no significant differences in the richness and evenness indices (Table 2, Pairwise Wilcoxon Rank Sum test, *p* > 0.05).

### 3.3. Soil Mycobiota Diversity

**Figure 4:**
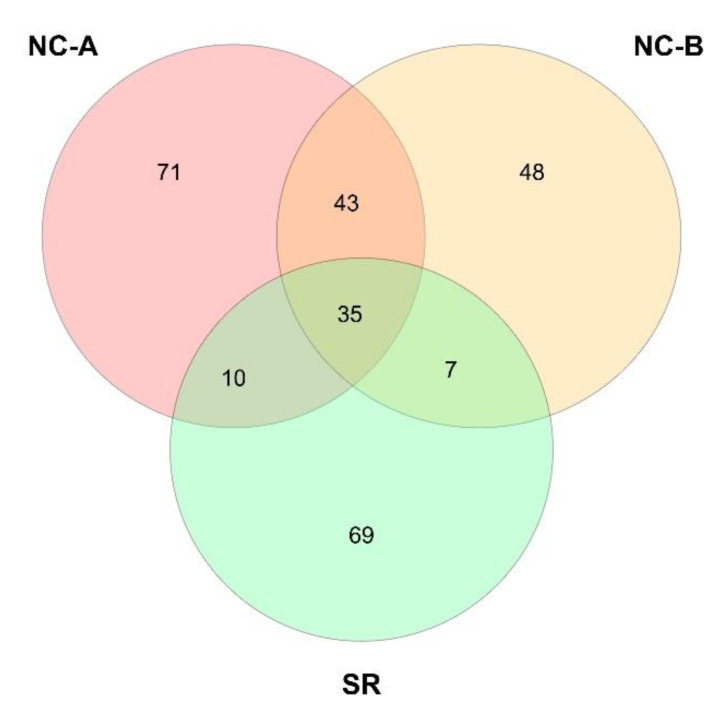
Venn’s diagram representing the number of OTUs identified at species level shared between NC-A, NC-B and SR plots.

Analyzing the OTUs identified at species level, each orchard plot had a distinctive fungal assemblage, when compared with the others (Figure 4). Indeed, 71 species were found uniquely in NC-A samples, 48 in NC-B samples and 69 in SR samples (Table S2). Predictably, quite a large number of species is shared between NC-A and NC-B samples (43 species), since the two areas are close to each other. Only 35 species were shared among the three plots, constituting the common mycobiota of these pear orchards (Table 3). Even if it comprises a contained number of species, the relative abundance of the OTUs of the common mycobiota amounts to 25% of all the found OTUs. In this common mycobiota, several members of Aspergillaceae, Nectriaceae and Mortiarellaceae were present. Regarding the fungal species which were unique for each site, though numerous, most of them were rare OTUs with very low abundance (<0.5%, Table S2). In SR samples, *Chaetomium homopilatum* (3.20%), *Leohumicola levissima* (2.44%), *Paraconiothyrium cyclothyrioides* (0.90%), and *Fusarium nisikadoi* (0.68%) were the most abundant unique OTUs. Among NC-A and NC-B samples, the only unique OTU with an abundance higher than 0.5% was *Inocybe curvipes* (1.78%).

**Figure 5:**
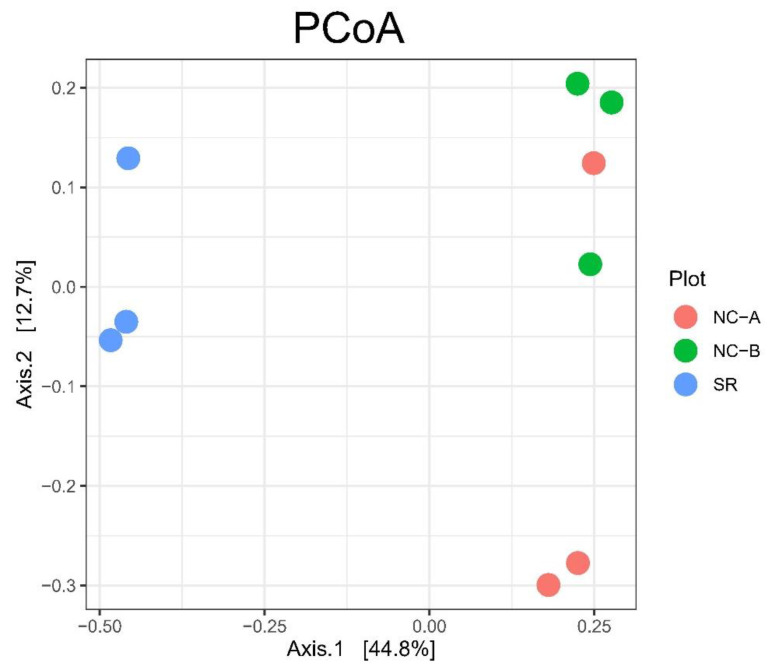
Principal coordinate analysis (PCoA) based on Bray–Curtis distances matrix of Illumina MiSeq sequencing fungal data of soil samples taken from pear orchards in Soracá (SR) and Nuevo Colón (steep slope plot at higher altitude: NC-A; flat plot at lower altitude: NC-B).

Based on the analysis of beta diversity using principal coordinate analysis (PCoA) on Bray–Curtis distance matrix, there was a statistically significant difference in the fungal communities between SR and NC samples (Figure 5, PERMANOVA, *p* < 0.05), while no significant difference could be detected between NC-A and NC-B samples (PERMANOVA, *p* > 0.05).

To assess how the mycobiota differed between the sites, the differential abundance of OTUs was determined using differential expression analysis (DESeq2 R package). Only OTUs identified at least at genus level with an abundance >1% in SR or NC plots were considered. According to these parameters, 13 fungal OTUs were significantly more abundant in SR plots (Table 4), while 11 OTUs were more abundant in NC plots (Table 5).

## 4. Discussion

The main finding of our study is a complete picture of bulk soil fungal biodiversity in high altitude pear orchards in the Colombian Andes, that will provide invaluable knowledge to the still developing fruit tree cultivation of the area and add new records to the ongoing discovery of soil fungal biodiversity in Colombia, especially for agricultural soils.

In this work, 194 fungal OTUs were identified at species level, 178 of which were categorized as microfungi. Out of these OTUs, 157 (79%) were new soil fungal records in Colombia, when compared with the cumulative review on Colombian soil microfungi of Landínez-Torres et al. [16]. The abundance of new records is a sign of how little is known of soil microfungi in Colombia and how crucial it is to carry on research in this field. At genus level, only 33% of the genera have been already found in Colombia [16]. Almost all of them were found in the Andean natural region, which is the most studied region and the one where our samples were collected. The most common genera found in our work and in at least 4 other studies were: *Mortierella* [28,42,43,44,45], *Fusarium* [28,42,45,46,47,48,49], *Humicola* [28,42,47,50], *Chaetomium* [28,42,47,48], *Clonostachys* [28,42,44,46,51], *Penicillium* [28,42,43,44,45,46,47,49,50,51,52,53,54,55,56], *Trichoderma* [42,44,45,46,51,55,56,57,58,59], *Mucor* [28,42,44,45,46,54,55], *Cladosporium* [28,42,46,47,49,59], and *Aspergillus* [28,42,43,45,46,47,48,49,50,51,53,55]. These genera are mostly ubiquitous in agricultural soils [60,61] and they can be considered as core fungi for the soils in the Andean region. In fact, species belonging to the above-mentioned genera (except *Trichoderma*) were found among the taxa shared between the three pear orchards here studied (Table 3).

In our study, Mortierellomycota was the second most abundant phylum and *Mortierella* was the most abundant genus amongst all. The high prevalence of this taxon was noticed also in peach and apple orchards’ soil in the Colombian Andean region [28]. *Mortierella*, then, seems to be an important taxon for Colombian agricultural soils, especially in the Andean region [16]. *Mortierella* spp. are saprotrophic fungi and very valuable decomposers in agricultural soils [62]. Together with species of *Aspergillus* and *Penicillium*, *Mortierella* spp. are the most abundant filamentous fungi in the soils around the world and they can be very promising plant-growth-promoters in agriculture [62,63]. As already noticed by Landínez-Torres et al. [28], members of *Mortierella* were numerically more abundant where the phosphorous concentration in soil was higher, that is, in our case, in NC samples, where organic fertilization was used. The concentration of *Mortierella* was especially high in the NC-B plot, that was at the foothill of the steep slope of NC-A plot, where probably the nutrients from fertilization were washed down by rainfall. This is in accordance with the observations of Li et al. [64], that linked the abundance of *Mortierella* with the long-term application of organic fertilizer.

From the sequencing data we found no evidence of the mycorrhizal phylum Glomeromycota in our bulk soil samples. Arbuscular mycorrhizal fungi (AMF) have been extensively studied in Colombia due to their fundamental ecological role, often in studies exclusively dedicated to this fungal group [65,66,67,68,69,70]. Indeed, *Glomus*, *Acaulospora* and *Rhizophagus* are among the most reported genera for Colombian fungi [16]. From previous comparable studies in Colombia, the presence of Glomeromycota in agricultural bulk soil is quite low (<1% of the total OTUs number) [28], but their total absence in our study was unexpected. We believe that a combination of different causes led to this result: the high concentration of phosphorous in the orchards’ soil could have hindered the growth of this phylum [71,72]; the molecular primers set that was used may not optimally capture Glomeromycota [73] or these fungi may not be so well represented in the ITS UNITE v.8.2 database. In order to investigate the last hypothesis, a random manual control of the obtained sequences, comparing them with the Mycobank database [74], detected that a few of the reads could actually be identified as Glomeromycota. As a consequence, we think that some of the biodiversity especially regarding this phylum could be hidden among the sequences that resulted as unassigned when compared with the database UNITE v.8.2. Due to the ecological importance of Glomeromycota in soil, future fungal community studies should adopt supplementary measures to make sure to detect the presence of this fungal phylum, such as the use of an additional primers set to target specifically Glomeromycota and the comparison with multiple fungal sequence databases.

Among the mycobiota shared in the three plots (Table 3), some species of *Mortierella* that were found are reported in the literature as fungi of bioprospective interest. *M. exigua* has potential as an agent of bioremediation against heavy metals [75], while *M. camargensis* and *M. amoeboidea* can both accumulate high concentrations of arachidonic acid in prospective bioenergy production [76]. Moreover, *M. amoeboidea* has shown herbicidal activities [77]. Three members of the Chaetomiaceae were also present in the common mycobiota: *Humicola olivacea*, *Chaetomium homopilatum*, and *H. nigrescens*. These species are often isolated from soil, compost or rotting plant materials [78,79]. Some strains of *Humicola* have shown potential as bio-organic fertilizers or as biocontrol organisms of plant diseases [80,81,82]. Moreover, *Solicoccozyma terrea*, *Fusarium solani* and *Exophiala radicis* were also found at a higher than 1% proportion in the common mycobiota. *Solicoccozyma terrea* is a basidiomycetous yeast commonly found in soil. It is known for its production of indole-3-acetic acid (IAA), which is the most common phytohormone occurring in plants, and it regulates various aspects of plant growth and development [83,84]. *Fusarium solani* is a common and ubiquitous soil species. It is usually associated with plant roots, but it can also be pathogenic for a wide variety of plants, such as peas, beans, potatoes, and many types of cucurbits [85], but no data were found on its pathogenicity on pear trees. Lastly, members of the *Exophiala* genus are mostly studied for their role as etiologic agents of disease in animals and humans [86,87], but they actually thrive in multiple habitats. In fact, they can also live saprophytically in bulk soil, biological crusts, rock surfaces, air, natural water masses, and rhizosphere [88,89,90,91]. In particular, strains of *Exophiala radicis* were found associated with roots both in Europe and in South America [91]. As concerns the presence of Zygomycota in the shared mycobiota, only *Mucor moelleri* and *Absidia anomala* were found in low abundances (0.23% and 0.04%, respectively) and they are both cosmopolitan saprotroph fungi inhabiting various environments [92,93].

The Venn diagram of shared OTUs identified at species level (Figure 4) highlights a high number of unique fungal OTUs for each site (Table S2). However, they were mainly rare OTUs (relative abundance < 0.5%), so these OTUs contribute to the general biodiversity and characterization of the orchard mycobiota without being the main characters of the community. Uniquely in SR samples, another strain of *Chaetomium homopilatum* was found (3.20%), then *Leohumicola levissima* (2.44%), a heat-resistant fungus already found both in cultivated and natural environments in the department of Boyacá (Colombia) [28], *Paraconiothyrium cyclothyrioides* (0.90%), an environmentally ubiquitous species that could turn into a human pathogen in immunocompromised patients [94], and the plant pathogen *Fusarium nisikadoi* (0.68%). The pioneer ectomycorrhizal species *Inocybe curvipes* was the only abundant unique OTUs (1.78) among NC-A and NC-B samples [95].

Among the OTUs with a significantly higher abundance in SR plots (Table 4), potentially phytopathogenic species were found, such as *Fusarium* sp. and *Cylindrocarpon* sp. These fungi can live saprophytically in soil but sometimes attack a wide variety of plants [85,96], so attention should be paid by the farmers of SR plots for the detection of possible symptoms of these pathogens. Moreover, two different species of *Clavaria* were detected, a basidiomycetous fungus reported as a saprotroph, decomposing leaf litter. Strains of *Clavaria* may also play a role as deep humic decayers [97]. The fruiting bodies of *Clavaria* are also of interest for their content in antioxidant compounds and essential trace elements, beneficial to human health [98].

Regarding the OTUs more abundant in NC plots (Table 5), three strains of *Mortierella* were found: *Mortierella alpina* (6,44%), *Mortierella gamsii* (3.44%), and an unidentified *Mortierella* sp. 2 (1.20%). As mentioned above, this abundance in NC plots as compared with SR plots could be related to the higher soil phosphorous concentration due to the use of organic fertilizer in NC plots. The most abundant OTUs in Table 5 is *Pseudaleuria* sp. (9.76%), belonging to the Pyronemataceae, whose members are often ectomycorrhizal symbionts [99]. Moreover, *Pseudaleuria* was found to be particularly abundant in healthy soils [100] and to have a negative correlation with the disease severity index of roots of *Pisum sativum* L. [101]. *Pseudallescheria fimeti* (5.23%), *Cladorrhinum* sp. (1.54%) and *Exophiala pisciphila* (1.09%) were also among the fungi that were more abundant in NC plots. Multiple fungi belonging to the *Pseudallescheria* genus were described as human pathogens, as agents of opportunistic infections [102]. However, they can live saprophytically in different environments; for example, *Pseudallescheria fimeti* was found in vermicompost [103]. *Cladorrhinum* species, on the other hand, are known as biocontrol agents [104], for example *Cladorrhinum flexuosum*, a wheat endophyte, can inhibit the growth of *Waitea circinata*, the causal agent of wheat root rot [105]. *Exophiala pisciphila* is an ascomycetous black yeast and can have positive symbiotic relationships with different plants. Strains of this fungus have managed to mitigate strawberry Fusarium wilt [106] and enhanced plant stress tolerance in heavy metals soils [107].

The fungi with bioprospective interest we found in these orchards are fundamental both for the description of the fungal community and for the possibility of using them in the future to improve the health of the pear tree orchard, limiting the use of phytochemicals. In fact, they could be isolated from soil, then cultivated in laboratory with the resulting spores re-introduced in soil or in compost [108]. This bio-augmenting and enrichment of the fungal soil community with native strains is important because it helps preserve the delicate equilibrium of the soil fungal biodiversity of an area [109,110].

From the literature, the study by Landínez-Torres et al. [28] is the most similar to our study, because we used the same technique to assess biodiversity in soil and our SR orchard is located in a neighboring area. In spite of these similarities, we found only 33 shared fungal species, a number comparable with the number of shared species among our three sites. Approximately half of these species belonged to Ascomycota, the most abundant phylum in both works, and they were several species of *Penicillium* and *Clonostachys*. Moreover, other ascomycete abundant common species were *Leohumicola levissima*, *Diaporthe columnaris*, *Metacordyceps chlamydosporia*, *Auxarthron umbrinum*. The only Basidiomycete in common was the yeast *Solicoccozyma terrea*. For the phylum Mortierellomycota, several species of *Mortierella* were shared, like *M. exigua*, which was especially abundant in all our samples, *M. elongata*, which was present only in NC samples, and *M. alpina* and *M. gamsii*. Finally, for Mucoromycota, *Absidia cylindrospora*, *Actinomucor elegans* and several species of *Mucor* were shared.

## 5. Conclusions

In conclusion, this work is an in-depth description of the bulk soil mycobiota found in two high-altitude pear orchards in the department of Boyacá (Colombia). This data will contribute to an environmentally friendly development of pear tree cultivation in the Colombian Andes, together with a socio-economic improvement for the population due to the more profitable culture. Our study also contributes to the increase of information about the agricultural soil fungal biodiversity of Colombia, a country with enormous potential in biodiversity discovery. Many of the identified fungal species are considered bioactive fungi, with promising activities as biocontrol agents, plant-growth promoters, and producers of valuable substances. Knowing about the presence of these fungi in Colombian soils may encourage further studies on their abilities and applications, leading to a more sustainable lifestyle.

## Figures and Tables

**Table 1:** Chemical analysis of soil samples taken from Soracá (SR) and Nuevo Colón (steep slope plot at higher altitude: NC-A; flat plot at lower altitude: NC-B). pear orchards (mean values ± standard deviation). * Significant at 0.05 probability level (*p* < 0.05), Kruskal–Wallis test with Bonferroni correction for multiple comparisons.

Plot	Sand	Silt	Clay	Soil texture	Moisture (105 °C)	pH	C org.
	(%)	(%)	(%)	(USDA)	(g/kg)		(%)
SR	36.6 ± 2.6	47.4 ± 2.3	15.9 ± 4.1	Loam	211.1 ± 13.9	5.4 ± 0.2 *	2.3 ± 0.1
NC-A	38.3 ± 4.5	44.4 ± 8.1	17.3 ± 3.6	Loam	213.2 ± 14.6	6.1 ± 0.3	3.2 ± 0.4
NC-B	39.3 ± 2.1	47.9 ± 1.4	12.8 ± 0.7	Loam	203.7 ± 6.0	6.6 ± 0.1 *	2.8 ± 0.3
**Plot**	**Organic matter**	**N tot**	**C/N**	**Ca**	**Mg**	**K**	**P**
	**(%)**	**(%)**		**(mg/kg)**	**(mg/kg)**	**(mg/kg)**	**(mg/kg)**
SR	4.0 ± 0.2	0.21 ± 0.01	11.1 ± 0.3	1540 ± 202	156 ± 36	355 ± 31	97.0 ± 10.0 *
NC-A	5.5 ± 0.7	0.25 ± 0.03	12.7 ± 0.1	2700 ± 400	144 ± 16	304 ± 27	209.1 ± 34.4
NC-B	4.9 ± 0.4	0.25 ± 0.02	11.5 ± 0.6	3060 ± 340	168 ± 13	347 ± 78	281.2 ± 23.6 *

**Table 2:** Richness (observed species) and diversity indices (Shannon and Simpson) based on Illumina MiSeq sequencing data (mean ± standard deviation) of soil samples taken from Soracá (SR) and Nuevo Colón (steep slope plot at higher altitude: NC-A; flat plot at lower altitude: NC-B).

	Observed	Shannon	Simpson
SR	201.67 ± 26.58	4.31 ± 0.33	0.97 ± 0.01
NC-A	222.33 ± 1.53	4.36 ± 0.04	0.97 ± 0.01
NC-B	180.33 ± 37.87	3.89 ± 0.49	0.93 ± 0.05

**Table 3:** List of OTUs identified at species level which were shared between the soil samples taken from Soracá (SR) and Nuevo Colón (steep slope plot at higher altitude: NC-A; flat plot at lower altitude: NC-B).

Species	Relative Abundance
*Mortierella exigua* Linnem.	6.69%
*Humicola olivacea* X.Wei Wang & Samson	2.13%
*Solicoccozyma terrea* (Di Menna) Yurkov	1.80%
*Chaetomium homopilatum* Omvik	1.47%
*Mortierella camargensis* W. Gams & R. Moreau	1.43%
*Fusarium solani* (Mart.) Sacc.	1.35%
*Exophiala radicis* Maciá-Vicente, Glynou & M. Piepenbr. var.1	1.30%
*Mortierella amoeboidea* W. Gams	1.20%
*Humicola nigrescens* Omvik	1.09%
*Solicoccozyma phenolica* (Á. Fonseca, Scorzetti & Fell) Yurkov	0.63%
*Bionectria rossmaniae* Schroers	0.49%
*Gibberella intricans* Wollenw. var.1	0.48%
*Exophiala radicis* Maciá-Vicente, Glynou & M. Piepenbr. var.2	0.44%
*Metacordyceps chlamydosporia* (H.C. Evans) G.H. Sung, J.M. Sung, Hywel-Jones & Spatafora	0.42%
*Thelonectria rubrococca* (Brayford & Samuels) Salgado & P. Chaverri	0.40%
*Clonostachys divergens* Schroers	0.38%
*Diaporthe columnaris* (D.F. Farr & Castl.) Udayanga & Castl.	0.37%
*Mortierella alpina* Peyronel	0.32%
*Cladosporium delicatulum* Cooke	0.29%
*Auxarthron umbrinum* (Boud.) G.F. Orr & Plunkett	0.27%
*Fusarium cuneirostrum* O’Donnell & T. Aoki var.1	0.25%
*Fusarium cuneirostrum* O’Donnell & T. Aoki var.2	0.23%
*Mucor moelleri* (Vuill.) Lendn.	0.23%
*Mortierella gamsii* Milko	0.16%
*Periconia macrospinosa* Lefebvre & Aar.G. Johnson	0.15%
*Exophiala bonariae* Isola & Zucconi	0.13%
*Ilyonectria robusta* (A.A. Hildebr.) A. Cabral & Crous	0.12%
*Aspergillus wentii* Wehmer	0.11%
*Penicillium virgatum* Nirenberg & Kwaśna	0.09%
*Gibberella intricans* Wollenw. var.2	0.07%
*Penicillium camemberti* Thom	0.07%
*Metarhizium marquandii* (Massee) Kepler, S.A. Rehner & Humber	0.06%
*Exophiala pisciphila* McGinnis & Ajello	0.05%
*Absidia anomala* Hesselt. & J.J. Ellis	0.04%
*Penicillium jensenii* K.W. Zaleski	0.03%

**Table 4:** List of OTUs with a significantly higher abundance in SR plots compared with NC plots (differential expression analysis based on the negative binomial distribution). The p-values shown are adjusted by false discovery rate (FDR, cut-off at 0.01).

OTUs	Relative Abundance SR	Relative Abundance NC	Adjusted *p*-Value
*Fusarium* sp. 1	8.47%	0.00%	5.74 × 10^−25^
*Chaetomium homopilatum* Omvik	4.81%	0.00%	5.25 × 10^−22^
*Leohumicola levissima* H.D.T. Nguyen & Seifert	3.62%	0.00%	9.34 × 10^−11^
*Cylindrocarpon* sp. 1	3.00%	0.00%	1.44 × 10^−4^
*Solicoccozyma* sp.	2.36%	0.00%	1.60 × 10^−14^
*Paraconiothyrium cyclothyrioides* Verkley	1.61%	0.00%	2.46 × 10^−11^
*Clavaria* sp. 1	1.34%	0.00%	3.22 × 10^−4^
*Fusarium nisikadoi* T. Aoki & Nirenberg	1.30%	0.00%	8.39 × 10^−10^
*Cylindrocarpon* sp. 2	1.15%	0.00%	1.60 × 10^−9^
*Clavaria* sp. 2	1.08%	0.00%	5.20 × 10^−4^
*Amaurodon* sp.	1.05%	0.00%	9.07 × 10^−4^
*Mortierella* sp. 1	1.00%	0.00%	1.53 × 10^−8^

**Table 5:** List of OTUs with a significantly higher abundance in NC plots compared with SR plots (differential expression analysis based on the negative binomial distribution). The p-values shown are adjusted by false discovery rate (FDR, cut-off at 0.01).

OTUs	Relative Abundance SR	Relative Abundance NC	Adjusted *p*-Value
*Pseudaleuria* sp.	0.08%	9.76%	5.86 × 10^−3^
*Mortierella alpina* Peyronel	0.00%	6.44%	5.90 × 10^−18^
*Fusarium* sp. 2	0.00%	5.95%	4.01 × 10^−19^
*Pseudallescheria fimeti* (Arx, Mukerji & N. Singh) McGinnis, A.A. Padhye & Ajello	0.00%	5.23%	2.64 × 10^−17^
*Solicoccozyma terrea* (Di Menna) Yurkov var.1	0.41%	4.98%	2.17 × 10^−4^
*Mortierella gamsii* Milko	0.00%	3.17%	2.38 × 10^−10^
*Cylindrocarpon* sp. 3	0.00%	2.48%	3.78 × 10^−8^
*Cladorrhinum* sp.	0.00%	1.54%	4.47 × 10^−11^
*Solicoccozyma terrea* (Di Menna) Yurkov var.2	0.00%	1.43%	1.44 × 10^−11^
*Mortierella sp. 2*	0.00%	1.20%	1.07 × 10^−12^
*Exophiala pisciphila* McGinnis & Ajello	0.00%	1.09%	7.04 × 10^−10^

## Data Availability

The data presented in this study are openly available in the NCBI Sequence Read Archive (SRA, https://www.ncbi.nlm.nih.gov/sra, accessed on 6 July 2021) under the BioProject number PRJNA748561.

## Supplementary Materials

The following are available online at www.mdpi.com/2079-7737/10/10/1002/s1, Table S1: identified OTU list with reads abundances divided according to the samples (SR1, SR2, SR3: Soracá samples 1, 2, 3, respectively; NC-A 1, 2, 3: Nuevo Colón, steep slope plot at higher altitude; NC-B 1, 2, 3 Nuevo Colón, flat plot at lower altitude); Table S2: List of OTUs identified at species level unique for each site (SR: Soracá, NC-A: Nuevo Colón, steep slope plot at higher altitude, NC-B: Nuevo Colón, flat plot at lower altitude).
